# Comparison of Quality of Life after Robotic, Video-Assisted, and Open Surgery for Lung Cancer

**DOI:** 10.3390/jcm12196230

**Published:** 2023-09-27

**Authors:** Nicole Asemota, Alessandro Maraschi, Savvas Lampridis, John Pilling, Juliet King, Corinne Le Reun, Andrea Bille

**Affiliations:** 1 Department of Thoracic Surgery, Guy’s Hospital London, Great Maze Pond, London SE1 9RT, UKcorinne.lereun@yahoo.fr (C.L.R.); 2 Division of Cancer Studies, King’s College London, Guy’s Hospital London, Great Maze Pond, London SE1 9RT, UK

**Keywords:** quality-of-life, robotic-assisted thoracoscopic surgery (RATS), symptoms, thoracotomy, video-assisted thoracoscopic surgery (VATS)

## Abstract

Post-operative quality of life (QOL) has become crucial in choosing operative approaches in thoracic surgery. However, compared to VATS and thoracotomy, QOL results post-RATS are limited. We compared QOL before and after RATS and between RATS, VATS, and thoracotomy. We conducted a retrospective review of lung cancer surgical patients from 2015 to 2020. Patients completed validated EORTC QOL questionnaires (QLQ-C30 and QLQ-LC13). Results were analysed using the EORTC Scoring Guide, with statistical analysis. A total of 47 (94%) pre- and post-RATS questionnaires were returned. Forty-two patients underwent anatomical lung resections. In addition, 80% of patients experienced uncomplicated recovery. All global and functional QOL domains improved post-operatively, as did most symptoms (13/19). Only four symptoms worsened, including dyspnoea (*p* = 0.017), with two symptoms unchanged. Of the 148 returned questionnaires for all approaches (open-22/VATS-79/RATS-47), over 70% showed a high pre-operative performance status. Most patients underwent anatomical lung resection, with only VATS patients requiring conversion (n = 6). Complications were slightly higher in RATS, with one patient requiring re-intubation. RATS patients demonstrated the highest global and functional QOL. Physical QOL was lowest after thoracotomy (*p* = 0.002). RATS patients reported the fewest symptoms, including dyspnoea (*p* = 0.046), fatigue (*p* < 0.001), and pain (*p* = 0.264). Overall, RATS results in a significantly better post-operative QOL and should be considered the preferred surgical approach for lung cancer patients.

## 1. Introduction

Lung cancer continues to be the most prevalent cancer globally, with surgery remaining the standard treatment for early-stage cases. Technological advancements have shifted surgical approaches from open procedures to minimally invasive surgeries, such as video-assisted thoracoscopic surgery (VATS) and robot-assisted thoracoscopic surgery (RATS) [1,2]. However, these two methods have significant differences. VATS has faced criticism for its limited manoeuvrability and reliance on a two-dimensional screen [1,2,3,4]. Consequently, RATS has gained attraction, addressing many VATS challenges by providing 360-degree articulation and a three-dimensional operating environment [1,2,5]. These advantages may offer RATS an oncological edge, facilitating improved nodal resection [1,6,7,8] and enabling more complex procedures, thus reducing VATS conversion rates [7,9].

The benefits of VATS compared to open surgery, including enhanced recovery and superior surgical outcomes, are well-established [1,2,6]. However, post-operative quality of life (QOL) is increasingly used to assess the true success of surgery. The VIOLET Trial [10] demonstrated that VATS led to a higher post-operative QOL and reduced adverse events compared to open surgery without compromising oncological outcomes. QOL comparisons between VATS and open surgery are abundant, consistently favouring VATS [1,4,6,10,11,12,13,14,15]. However, QOL comparisons with RATS are not as strong currently, and tripartite comparisons of all three approaches even less so. Such comparisons are frequently limited by the lack of QOL data in RATS patients and disagreement amongst the minimal RATS QOL data available. Considering the substantial learning and financial investments required for RATS, comprehensive reviews of QOL in RATS patients are increasingly necessary.

This study will compare QOL before and after RATS and assess QOL following VATS, RATS, and open surgery in lung cancer patients after thoracic surgery.

## 2. Materials and Methods

We conducted a retrospective study of local patients. Patients were eligible for inclusion if they were older than 18 years, had a diagnosis of primary or secondary lung cancer, and underwent surgical resection for lung cancer at our institution between 2015 and 2020. Additional inclusion criteria were being alive without evidence of recurrence at the time of the study and not receiving adjuvant chemotherapy or radiation therapy at the time of QOL questionnaire completion. The surgical approaches included were thoracotomy (open surgery), VATS, and RATS. Acceptable types of lung resection were lobectomy, bilobectomy, segmentectomy, and wedge resection.

Patients were excluded if they died prior to study initiation, had recurrence of lung cancer, were receiving adjuvant therapy at the time of QOL assessment, underwent lung surgery for benign indications, or had surgery performed at another institution.

Eligible patients completed two QOL questionnaires designed by the European Association for Research and Treatment for Cancer (EORTC): QLQ-C30, assessing general QOL after cancer treatment, and QLQ-LC13, evaluating lung cancer-specific symptoms [16,17]. The primary endpoints of this study were changes in QOL amongst RATS patients before and after surgery, and QOL differences between RATS, VATS, and thoracotomy patients post-operatively. Secondary endpoints included differences in complication rates, ICU admission, conversion rates, and length of hospital stay.

**Figure 1:**
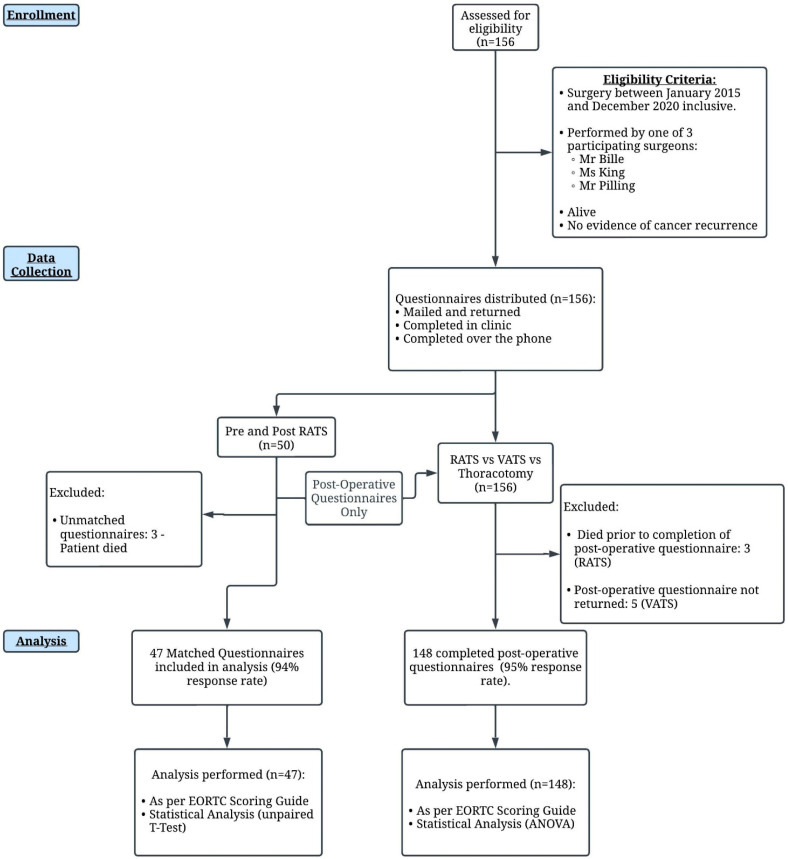
Overview of Methodology.

A pictorial summary of the methods is demonstrated in Figure 1.

This study conformed to the ethical principles defined in the Declaration of Helsinki of 1964 and all subsequent revisions, and it was approved by the relevant committee of our institution as a clinical audit project (Number 7753). Written informed consent was obtained from all participants prior to inclusion.

### 2.1. Pre- and Post-RATS

We included only matched pre- and post-operative RATS questionnaires. The pre-operative questionnaires for RATS patients were completed in the outpatient clinic during the preadmission appointment, along with an information sheet explaining the purpose of the study. The post-operative questionnaires for all groups were administered during follow-up appointments in clinic, through postal mailings, or over the telephone by the study authors. Along with the questionnaire, patients received an information sheet describing the study and instructions for completing the survey. Completed questionnaires were returned to the study authors either in-person during a clinic visit, via post, or the answers were documented by the study authors during telephone administration. Questionnaire results were analysed according to the EORTC Scoring Guide. We categorised QLQ-C30 results into Global Health Status, Symptoms, and Functional QOL, further subdividing functional QOL into five domains: Physical, Role, Emotional, Cognitive, and Social QOL. The QLQ-LC13 questionnaire focused solely on lung symptoms.

We generated two scores for all domains: a raw score (RS), the average of scores from all contributing questions, and a final, standardised score (FS), after applying a linear transformation formula to the RS. The RS was calculated as: Raw Score = (I1 + I2 + … + In)/n, with higher scores indicating better Global QOL and worse functional QOL/Symptoms. The FS was calculated as: Final Score = ((Rawscore − 1)/range) × 100 for Global Health and Symptom Scales; and Final Score = (1 − (Rawscore − 1)/range) × 100 for Functional Scales. Higher Scores indicate better Global and Functional QOL and worse symptomatology. Range = Maximal–minimal possible question score. (Global Health questions = 6, All other questions = 3). 

### 2.2. RATS, VATS, and Thoracotomy

We compared post-operative data from RATS patients against previously collected post-operative QOL data from patients who had undergone VATS and open surgery for lung cancer. We used the same questionnaires and analysis methods for all groups. Patient characteristics were described as above. 

### 2.3. Statistical Analysis

Analysis was performed using the Stata software version 14.2 (College Station, TX, USA). Population characteristics were analysed using summary statistics (mean and standard deviation for continuous variables; frequency and percentage for categorical variables). RATS pre- and post-operative scores were compared using unpaired T tests. RS and FS between RATS, VATS, and thoracotomy were compared using one-way analysis of variance. A *p*-value of <0.05 was deemed to be statistically significant. 

## 3. Results

### 3.1. Pre- and Post-RATS

Forty-seven patients were included in the final analysis (Figure 1). Patient characteristics are described in Table 1.

Most patients were female (68%), with an average age of 69 years. Most patients had a performance status (PS) of 0 or 1. Twenty-nine (61.7%) were ex-smokers, with a median of 21 pack years. Almost all patients had co-morbidities, particularly cardiac (n = 26; 55%) and lung (n = 12; 25.5%) diseases. A total of 43 patients (91%) underwent anatomical lung resections, most commonly lobectomy (70.2%, n = 33). All RATS procedures were performed by a single surgeon (AB). All four non-anatomical wedge resections performed were for lung metastases.

In total, 82.9% of patients had an uncomplicated recovery [Clavien–Dindo = 0 (n = 30) or 1 (n = 9)]. The most common complication was prolonged air leak (>7 days) and pneumonia, occurring in five patients (10.6%) and four patients (8.5%), retrospectively. Two patients (4.3%) developed COVID-19. One patient (2.1%) had a Clavien–Dindo score of 4, experiencing pneumonia and respiratory failure that required admission to critical care and re-intubation. Post-operative questionnaires were completed with an average interval period of 7 months (range, 5–9). Notably, 83.3% of patients returned to baseline health within 2 to 3 months. 

#### Quality of Life Results

**Figure 2:**
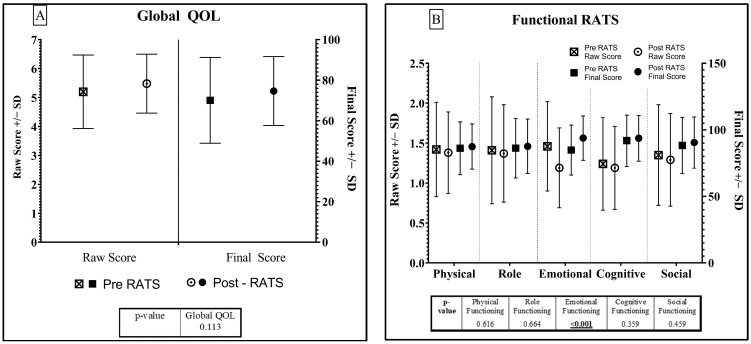
Global (**A**) and Function (**B**) QOL Results—Pre- and Post-RATS. Standard deviation shown in bars.

**Figure 3:**
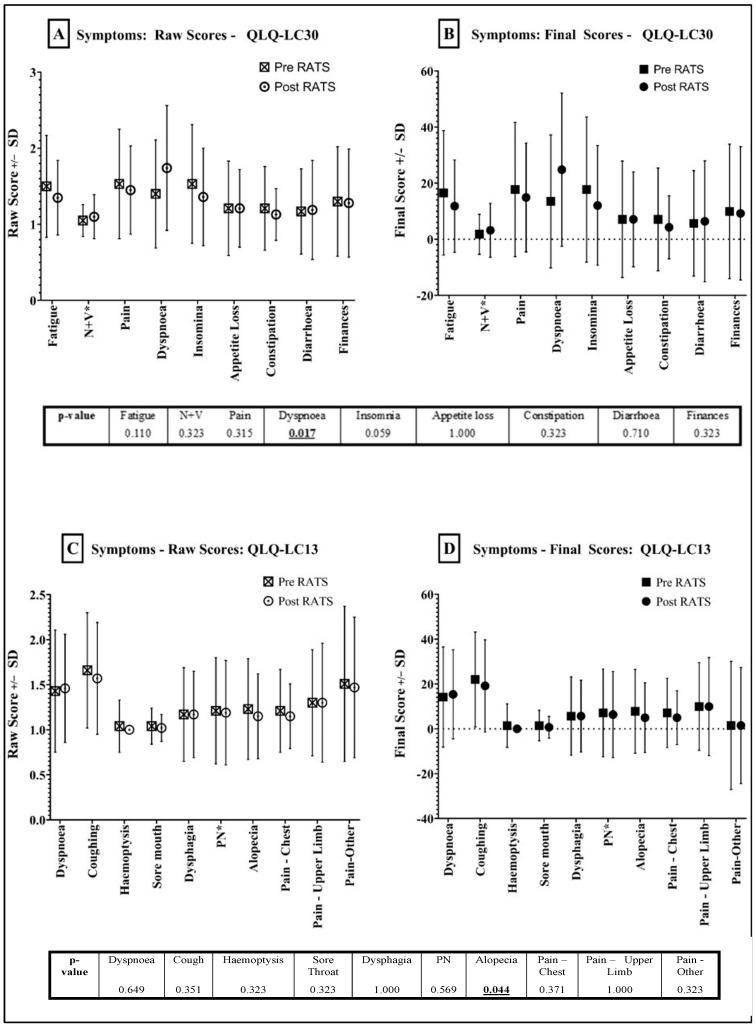
Symptom Scores—Pre- and Post-RATS. QLQ-C30 Results = (**A**) (RS) and (**B**) (FS). LC13 Results = (**C**) (RS) and (**D**) (FS). Standard deviation shown in bars.

The complete QOL results are displayed in Figure 2 and Figure 3 and Supplemental Table S1. Global QOL (Figure 2A) appears to have improved post-RATS (*p* = 0.113). There was an improvement in functional QOL in all five domains post-RATS, with a statistically significant improvement in emotional functioning (FS: increase of (+) 9, *p* < 0.001) (Figure 2B). Importantly, patients demonstrated a mild improvement in physical health post-RATS (FS: +1.42).

Symptoms assessed in the QLQ-C30 questionnaire (Figure 3A,B) showed an improvement in six out of nine symptoms, although these were not statistically significant. Nausea/vomiting and diarrhoea appeared to worsen post-RATS (FS: +1.42 and +0.71, respectively). Results from QLQ-LC13 (Figure 3C,D) were similar, with an improvement in most symptoms, but statistically significant only in alopecia (FS decrease of (−) 2.84, *p* = 0.044). 

Dyspnoea and pain were the only QOL measures that appeared in both questionnaires. In both questionnaires, dyspnoea appeared to worsen post-RATS. However, this was only statistically significant in QLQ-C30 (FS: +11.34, *p* = 0.017), with a much smaller FS increase of 1.19 in QLQ-LC13. In both questionnaires, pain was overall lower post-RATS, although not statistically significant.

### 3.2. RATS vs. VATS vs. Thoracotomy

We included 148 patients in the analysis: VATS, n = 79; RATS, n = 47; and open surgery, n = 22. Patient characteristics are described in Table 2.

VATS patients were, on average, 5.8 years older (75.6 years) than thoracotomy (68.2 years) and RATS (69.8 years) patients (*p* < 0.001). Sex was more evenly split in VATS (58.2% female) and thoracotomy patients (50% each) than in RATS patients (68.1% female). A PS of 0 or 1 was reported in over 70% of all groups. Most patients were ex-smokers (median of 21 pack years). 

Only 8% of all patients had no additional co-morbidities (VATS, n = 9; thoracotomy, n = 3). The most common co-morbidities in all groups were pulmonary (thoracotomy, 31.8%; VATS, 35.4%; RATS, 25.5%) and cardiac (thoracotomy, 50%; VATS, 68.4%; RATS, 55.3%) diseases. Previous lung cancer was most common in the VATS group (31.6%). The average pre-operative staging was higher in the thoracotomy group (Stage II and III at 32% each) compared to the VATS and RATS group (Stage I—77% (VATS) and 68% (RATS)). 

Most patients underwent anatomical lung resection (>80% for all groups, *p* = 0.251). AB, JK, and JP all contributed to VATS and thoracotomy procedures. Segmentectomies were at least five times more common in VATS and RATS patients compared to thoracotomy. Conversion to thoracotomy occurred only in VATS patients (n = 6, 7.6%). Inpatient complications occurred at a higher proportion after RATS (36.2% vs. 26.6%—VATS vs. 27.3%—thoracotomy, *p* = 0.504). Hospital-Acquired Pneumonia (HAP) was the most common complication in all groups. Although more VATS patients developed HAP (n = 12), this was proportionally smaller compared to thoracotomy (15.2% vs. 18.2%), but almost double compared to RATS (15.2% vs. 8.5%) patients. Most patients had Clavien–Dindo scores of 0 or 1. Only 1 RATS patient had a score of 4 (described above).

#### Quality of Life Results

**Figure 4:**
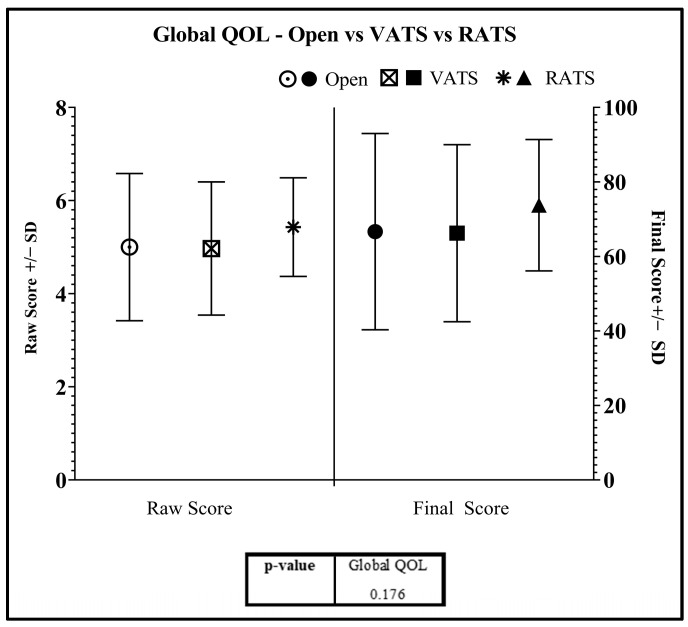
Global QOL Scores—RATS vs. VATS vs. Open. Standard deviation shown in bars.

**Figure 5:**
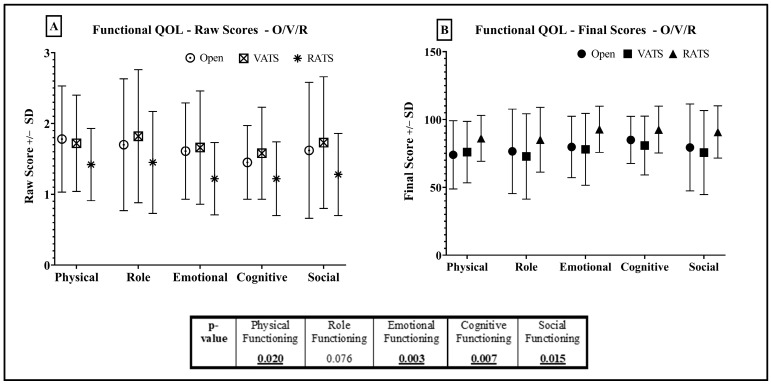
Functional QOL—RATS (R) vs. VATS (V) vs. Open (O). Raw Scores (RS) = (**A**), Final Scores (FS) = (**B**). Standard deviation shown in bars.

**Figure 6:**
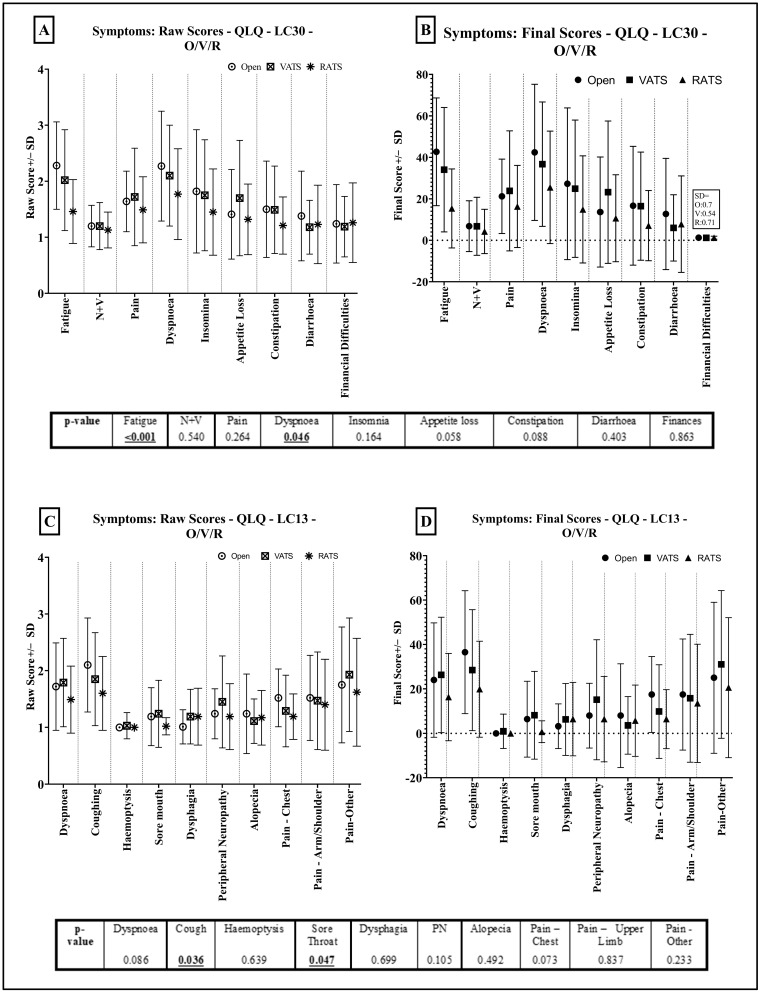
Symptom Scores—RATS (R) vs. VATS (V) vs. Open (O). QLQ-C30 Results = (**A**) (RS) and (**B**) (FS). LC13 Results = (**C**) (RS) and (**D**) (FS). Standard deviation shown in bars.

All QOL results are shown in Figure 4, Figure 5 and Figure 6 and Supplement Table S2. Questionnaires were completed with an average interval period of 7 months amongst RATS patients and 10 months amongst VATS and open patients. Global QOL (Figure 4) was equal between thoracotomy and VATS patients (FS: 66.67 and 66.24), but higher amongst RATS patients (FS: 73.76, *p* = 0.176).

Functional QOL (Figure 5) was highest amongst RATS patients in all subdomains. FS was similar between open and VATS patients in all subdomains. VATS patients had the lowest scores in all sub-domains, except physical functioning where thoracotomy patients were lowest (FS:73.94—open vs. 79.95—VATS and 86.1—RATS, *p* = 0.02). Differences in all sub-domains, except role functioning, were statistically significant.

RATS patients were least symptomatic in almost all QLQ-C30 domains (Figure 6A,B), with statistically significant results in fatigue (*p* < 0.001) and dyspnoea (*p* = 0.046). VATS patients reported the least diarrhoea and least financial difficulties (*p* = 0.863) after surgery.

QLQ-LC13 lung-specific symptoms were low, with an average RS of <2 in all patients (Figure 6C,D). RATS patients were less symptomatic in all domains except alopecia (VATS patients with lowest FS: 3.56 vs. 7.94—thoracotomy, 6.38—RATS) and dysphagia (thoracotomy patients with lowest FS—3.17 vs. 6.22—VATS and 6.38—RATS). Coughing and sore throat were the only statistically significant scores (*p* = 0.036 and *p* = 0.047, respectively), with RATS patients the least symptomatic. 

Dyspnoea scores appeared to differ between questionnaires. RATS patients reported the lowest dyspnoea across both questionnaires, but with a larger and more statistically significant difference in QLQ-C30 (Figure 6A,B) compared to non-RATS patients than in the QLQ-LC13 (Figure 6C,D). RATS patients reported the least overall pain in both questionnaires. VATS patients reported the highest overall QLQ-C30 pain score (*p* = 0.264) (Figure 6A,B). Area-specific pain, in QLQ-LC13 (Figure 6C,D), was worst amongst thoracotomy patients in the arm/shoulder (*p* = 0.837) and chest (0.073), with only pain elsewhere appearing worse amongst VATS patients (*p* = 0.233).

## 4. Discussion

The present study provides robust evidence that QOL is higher amongst RATS patients, both in direct comparison before and after surgery, and when compared with VATS and open surgery. This finding corroborates the existing literature that demonstrates improved QOL among RATS patients [7,14,18,19].

However, notable discrepancies exist in the assessment of QOL in RATS patients within the literature [2,8,20]. These inconsistencies arise primarily from variations in study protocols, such as the surgical approaches compared (RATS only [2,19] vs. VATS [5,8,10,20] or vs. thoracotomy [7,19]) and the substantial differences in patient numbers. The present study addresses these limitations by utilising a single cohort to examine temporal changes in QOL in RATS patients while comparing them to VATS and thoracotomy patients. Moreover, the study benefits from being conducted at a single institution with a single surgeon (AB) performing all RATS operations, thereby mitigating the influence of the surgical learning curve for robotic surgery [7] and variable post-operative protocols, such as pain control. Consequently, our results suggest that the observed improvements in QOL are more likely attributable to RATS rather than alternative factors.

To the best of our knowledge, this study is the only clinical investigation that examines QOL in VATS, RATS, and thoracotomy patients simultaneously. Although recent meta-analyses and systematic reviews have attempted similar three-way comparisons, they have been hindered by the scarcity of the RATS QOL literature [6]. As a result, many studies group RATS and VATS into a ‘minimally invasive’ category, comparing them against thoracotomy [1,18]. This approach fails to demonstrate the specific superiority of RATS, including over VATS. Therefore, the present study is essential in illustrating the QOL differences between all three approaches independently.

Existing evidence suggests that the QOL benefits of RATS decrease over time, with a return to baseline after four months [18,21]. However, our study found that RATS patients maintained their higher QOL scores after seven months, indicating that these benefits may persist for a longer duration [18].

Prior studies have suggested that patients with a higher pre-operative PS are less symptomatic pre-operatively and thus exhibit higher overall QOL scores [14]. In our cohort, significant differences in QOL were observed across all three approaches despite over 75% of all patients having high PS scores (0 or 1). Therefore, as QOL results were different despite a largely similar PS amongst all patients, a pre-operative PS is unlikely to have had a significant impact in our results. However, further studies directly assessing PS on QOL are required. 

When compared to pre-operative assessments and non-RATS patients post-operatively, post-operative functional QOL was highest among RATS patients. Multiple studies have emphasised the advantages of minimally invasive surgery on physical functioning [13,14,20]. In our study, we observed no change in physical functioning before and after RATS surgery. This may be attributed to patient selection, as most RATS patients had a high PS and minimal symptoms pre-operatively, which may have rendered them less likely to notice significant changes post-operatively. However, RATS patients demonstrated a significantly higher physical QOL compared to VATS and thoracotomy patients. Thoracotomy patients, likely due to the larger incision, exhibited the lowest physical functioning QOL, which is consistent with the existing literature [13,14,20]. Emotional functioning was found to be significantly lower after RATS in both parts of the study. This supports current evidence and may be attributed to the smaller scar, reduced hospital stay, and enhanced post-operative recovery associated with RATS, leading to improved mental health.

Overall, symptoms were fewer among patients who underwent RATS. Pain was significantly lower in RATS patients compared to VATS and thoracotomy patients, both in our study group and within the current literature [6,13,18,20,21]. Nausea, vomiting, and constipation were also lowest in RATS patients. These findings may be partly attributed to side effects from post-operative pain medications, especially nausea and constipation from opioid use [6,13,18,20,21]. As such, it is plausible that the reduced pain experienced by RATS patients led to lower opioid use, and subsequently fewer drug-related side effects [7,22]. Interestingly, VATS patients reported more pain than thoracotomy patients, despite having a smaller incision. This might be due to the increased pressure from leverage exerted on the rib and intercostal bundle, [7] a phenomenon less noted in RATS (due to superior articulation) and open surgery. 

Dyspnoea is an important symptom amongst lung cancer patients. Overall, post-operative dyspnoea scores were worse in RATS patients compared to the baseline, but still lower when compared to non-RATS patients. The current literature suggests that patients with a higher pre-operative PS may report worse shortness of breath (SOB) symptoms post-operatively [14] as these patients will have had minimal pre-operative dyspnoea, will be less tolerant of small SOB changes, and may over-estimate the extent of change when asked. This may explain the worsening SOB post-RATS compared to the baseline in our cohort, in whom the pre-operative PS was high for the majority of RATS patients. However, this does not account for the clear dyspnoea difference noted amongst VATS, RATS, and thoracotomy patients despite a relatively equal pre-operative PS. Ultimately, further studies utilising a single dyspnea evaluation are needed to obtain a definitive answer [18,20].

A challenge in comparing the QOL literature lies in the numerous assessment methods available [4,7,23]. In this study, we used the highly validated EORTC questionnaires for the assessment of QOL [21,24] and, specifically, the QLQ-LC13, which is tailored to lung cancer patients. Additional questionnaires used in the literature include the 12-item Short Form Survey (SF12) [22], 36-item Short Form Survey (SF36) [12], and VAS pain score [4]. However, to our knowledge, the EORTC questionnaires are the most commonly used and validated QOL questionnaires [11,13,14,18,20,25]. Despite their widespread use, these questionnaires are not exempt from error, as demonstrated by the significant disparity when comparing dyspnea scores, the only identical symptom present on both questionnaires. Similar studies on QOL have also shown conflicting results within the same population group [20]. This underlines the subjective nature of questionnaires and the need for increased QOL data to validate results. 

This study has some limitations. First, the QLQ-LC13 questionnaire has been recently updated to include questions specifically related to QOL after lung cancer surgery [26]. However, we used the older version of the QLQ-LC13 to facilitate the comparison of RATS results with pre-collected VATS and thoracotomy data that utilised the original LC13 questionnaire. Future QOL studies could consider using the updated questionnaire [6,27]. Second, the number of patients per group was unequal, with the number of thoracotomy patients (n = 22) being less than a third of those in the VATS group (n = 79). This was unavoidable as VATS is the predominant approach in most institutions. In future studies, equal, propensity-matched groups would allow for a more accurate comparison of QOL.

A key limitation of this study was the lack of pre-operative QOL data for the VATS and thoracotomy groups. Without this baseline data, we could not definitively determine if the surgical approaches had equivalent impacts on QOL compared to each patient’s pre-operative status. While we prospectively obtained matched pre- and post-operative QOL assessments for RATS patients, the retrospective nature of the VATS and thoracotomy data precluded gathering pre-operative QOL information. The lack of baseline QOL data for all groups is a limitation, as we cannot exclude the possibility that pre-operative QOL variability influenced the differences seen post-operatively. Future prospective studies should incorporate pre-operative QOL assessments for all surgical groups to better evaluate the impact of each approach compared to the baseline.

Another recognised limitation in this study is the different timeframes for post-operative assessment between groups, with RATS post-op questionnaires completed at 7 months, and the VATS and thoracotomy patients completed at 10 months. It is possible that the QOL for RATS patients may have continued to improve further in the 3-month difference. Standardised timeframes would have strengthened our results, likely further highlighting the higher QOL for RATS compared to VATS and thoracotomy. Lastly, the QOL questionnaires are inherently subjective and specific to the patient cohort, making it challenging to generalise these results to the broader population. Further studies on QOL are needed for a more accurate assessment of post-operative QOL.

## 5. Conclusions

In conclusion, RATS appears to result in a minimal impact on quality of life when compared to pre-operative QOL and significantly improved QOL post-operatively when compared directly to VATS and open surgery in many categories, particularly in functional QOL. RATS demonstrated improved dyspnoea and reduced post-operative pain compared to VATS and thoracotomy. Therefore, RATS is likely to become the preferred operative approach, especially if QOL, length of stay (LOS), and operative outcomes can be balanced against its cost.

The findings of this study contribute to the growing body of evidence supporting the use of RATS in surgical practice, while highlighting the need for further research to validate and expand upon these results. By carefully considering the limitations and challenges associated with QOL assessments and addressing them in future studies, researchers can continue to advance our understanding of the impact of different surgical approaches on patient outcomes. Ultimately, these insights will help guide clinical decision making and ensure that patients receive the most effective and appropriate care for their individual needs.

## Figures and Tables

**Table 1:** Patient Characteristics amongst RATS patients.

	(N = 47)	
Age (Median)		69.8 ± 9.1
Gender	Male	15 (34.0%)
	Female	32 (68.1%)
PS ECOG	0	12 (25.5%)
	1	23 (48.9%)
	2	12 (25.5%)
Smoking status	Non smoker	10 (25.5%)
	Ex−smoker	29 (61.7%)
	Smoker	8 (17.0%)
Comorbidities	Pulmonary	12(25.5%)
	Cardiac	26 (55.3%)
	Previous cancer	11 (23.4%)
	Of which is primary lung cancer	2 (4.3%)
	Nil	5 (10.6%)
Procedure	Lobectomy	33 (70.2%)
	Bi lobectomy	0
	Segmentectomy	10 (21.3%)
	Wedge resection	4 (8.5%)
Number of Ports	4	47 (100%)
Operating Time:		
Mean (±SD), mins	110.8 (±38.8)	−
Median (IQR), mins	105 (41)	−
Pre-Operative Staging	IA1	4 (8.5%)
	IA2	15 (31.9%)
	IA3	12 (25.5%)
	IB	1 (2.1%)
	IIA	2 (4.3%)
	IIB	2 (4.3%)
	IIIA	2 (4.3%)
	IVB ^1^	1 (2.1%)
	Secondary Lung Metastases	7 (14.9%)
	No Pre-op Staging	1 (2.1%)
Post-Operative Staging	IA1	2 (4.3%)
	IA2	13 (27.7%)
	IA3	10 (21.3%)
	IB	6 (12.8%)
	IIA	1 (2.1%)
	IIB	5 (10.6%)
	IIIA	2 (4.3%)
	IIIB	1 (2.1%)
	No Staging (Secondary Metastasis)	7 (14.9%)
Post-Operative Histology	Adenocarcinoma	33 (70.2%)
	Squamous Cell Carcinoma	3 (6.4%)
	Metastasis	7 (14.9%)
	Carcinoid	4 (8.5%)
Complications	Inpatient complications	17 (36.2%)
	Of which:	
	COVID	2 (4.3%)
	Prolonged AL (>7 days)	5 (10.6%)
	AF	3 (6.4%)
	Atelectasis/sputum plug/bronchoscopy	3 (6.4%)
	Hospital Acquired Pneumonia (HAP)	4 (8.5%)
	Pleural effusion/empyema	2 (4.3%)
	Pneumothorax—new drain insertion	1 (2.1%)
Clavien–Dindo	0	30 (63.8%)
	1	9 (19.2%)
	2	5 (10.6%)
	3	0
	3a	2 (4.3%)
	4a	1 (2.1%)

^1^ Patient who was originally staged as M1C and restaged after chemotherapy and immunotherapy with residual disease in the lung and no evidence of metastatic disease.

**Table 2:** Patient Characteristics amongst thoracotomy, VATS, and RATS patients.

		Thoracotomy (N = 22)	VATS (N = 79)	RATS (N = 47)	*p*-Value
Age		68.2 ± 8.8	75.6 ± 9.6	69.8 ± 9.1	<0.001
Gender	Male	11 (50.0%)	33 (41.8%)	15 (31.9%)	0.32
	Female	11 (50.0%)	46 (58.2%)	32 (68.1%)	
PS ECOG	0	6 (27.3%)	15 (19.0%)	12 (25.5%)	0.86
	1	11 (50.0%)	45 (57.0%)	23 (48.9%)	
	2	5 (22.7%)	19 (24.0%)	12 (25.5%)	
Smoking Status	Non-Smoker	2 (9.1%)	9 (11.4%)	12 (25.5%)	0.24
	Ex-smoker	16 (72.7%)	58 (73.4%)	27 (57.5%)	
	Smoker	4 (18.2%)	12 (15.2%)	8 (17.0%)	
Co-morbidities	Pulmonary	7 (31.8%)	28 (35.4%)	12 (25.5%)	0.51
	Cardiac	11 (50.0%)	54 (68.4%)	26 (55.3%)	0.17
	Renal	−	4 (5.1%)	1 (2.1%)	0.57
	Previous cancer	6 (27.3%)	25 (31.6%)	11 (23.4%)	0.61
	Previous primary lung cancer	2 (9.1%)	2 (2.5%)	2 (4.3%)	0.29
Pre-Operative Staging	IA1	0 (0%)	12 (15.2%)	4 (8.5%)	
	IA2	2 (9.1%)	27 (34.2%)	15 (31.9%)	
	IA3	1 (4.5%)	8 (10.1%)	12 (25.5%)	
	IB	2 (9.1%)	14 (17.7%)	1 (2.1%)	
	IIA	1 (4.5%)	4 (5.1%)	2 (4.3%)	
	IIB	6 (27.3%)	8 (10.1%)	2 (4.3%)	
	IIIA	4 (18.2%)	0	2 (4.3%)	
	IIIB	2 (9.1%)	1 (1.3%)	0	
	IIIC	1 (4.5%)	0	0	
	IV	3 (13.6%)	0	1 (2.1%)	
	Secondary Metastasis		3 (3.8%)	7 (14.9%)	
	No Staging (No Pre-op Staging)		2 (2.5%)	1 (2.1%)	
Procedure	Lobectomy	15 (68.2%)	50 (63.3%)	33 (70.2%)	
	Pneumonectomy	2 (9.1%)	0	0	
	Segmentectomy	2 (9.1%)	17 (21.5%)	10 (21.3%)	
	Wedge resection	3 (13.6%)	12 (15.2%)	4 (8.5%)	
Number of Ports	3	−	79 (100%)	0	
	4	−	0	47 (100%)	
Operating Time:					
Mean (+/−SD), mins		143.2 (±38.4)	116.1 (±32.2)	110.8 (±38.8)	
Median (IQR), mins		142 (60)	120 (51.25)	105 (41)	
Conversion	Yes	N/A	6 (7.6%)	0	
	No	N/A	73 (92.4%)	47 (100%)	
Final Staging	IA1	0	4 (5.1%)	2 (4.3%)	
	IA2	0	18 (22.8%)	13 (27.7%)	
	IA3	0	9 (11.4%)	10 (21.3%)	
	IB	3 (13.6%)	17 (21.5%)	6 (12.8%)	
	IIA	2 (9.1%)	5 (6.3%)	1 (2.1%)	
	IIB	5 (22.7%)	11 (13.9%)	5 (10.6%)	
	IIIA	5 (22.7%)	6 (7.6%)	2 (4.3%)	
	IIIB	2 (9.1%)	1 (1.3%)	1 (2.1%)	
	0 (no staging)	1 (4.5)	0	0	
	Secondary Metastasis	4 (18.2%)	5 (6.3%)	7 (14.9%)	
	Benign Disease	0	1 (1.3%)	0	
	Carcinoid	0	1 (1.3%)	0	
	TNM Staging Not Applicable ^1^	0	1 (1.3%)	0	
Complications	In-hospital complications	6 (27.3%)	21 (26.6%)	17 (36.2%)	0.50
	COVID	0	0	2 (4.3%)	0.22
	Prolonged air leak (>7 days)	2 (9.1%)	3 (3.8%)	5 (10.6%)	0.23
	AF	0	9 (11.4%)	3 (6.4%)	0.24
	Airway complications	1 (4.5%)	4 (5.1%)	3 (6.4%)	>0.99
	HAP	4 (18.2%)	12 (15.2%)	4 (8.5%)	0.48
	Pleural effusion/empyema	0	1 (1.3%)	2 (4.3%)	0.73
	Surgical emphysema	0	2 (2.5%)	0	0.66
	Pneumothorax—new drain insertion	0	1 (1.3%)	1 (2.1%)	>0.99
Clavien–Dindo	0	16 (72.7%)	58 (73.4%)	30 (63.8%)	0.083
	1	2 (9.1%)	3 (3.8%)	9 (19.1%)	
	2	3 (13.6%)	15 (19%)	5 (10.6%)	
	3	1 (4.5%)	2 (2.5%)	0	
	3a	0	1 (1.3%)	2 (4.3%)	
	4a	0	0	1 (2.1%)	

^1^—Final Histology: Angiosarcoma; therefore, Lung TNM staging not applicable.

## Data Availability

The data presented in this study are available on request from the corresponding author. The data are not publicly available due to privacy concerns.

## Supplementary Materials

The following supporting information can be downloaded at: https://www.mdpi.com/article/10.3390/jcm12196230/s1, Table S1: Full QOL Results (Pre and Post RATS), Table S2: Full QOL Results (RATS vs. VATS vs. Thoracotomy).
