# Supplementary material for: Comparison of Quality of Life after Robotic, Video-Assisted, and Open Surgery for Lung Cancer
Source: J Clin Med. 2023 Sep 27;12(19):6230. doi: 10.3390/jcm12196230 (PMC10573228; doi:10.3390/jcm12196230)
Supplement: Supplementary file 1 [file jcm-12-06230-s001.zip › jcm-2581460-supplementary.pdf]

Table S1: Full QOL Results (Pre and Post RATS).

Scale	Score	Before RATS	After RATS	p-value
**QLQ-LC30**				
**GLOBAL HEALTH STATUS / QOL**				
**Global Health Status / QOL (QL2)**	Raw score<br>Final Score	5.20 +/- 1.27<br>70.04 +/- 21.19	5.48 +/- 1.02<br>74.65 +/- 17.02	0.113
**FUNCTIONAL SCALES**				
**Physical Functioning (PF2)**	Raw score<br>Final Score	1.42 +/- 0.59<br>86.10 +/- 19.80	1.38 +/- 0.51<br>87.38 +/- 17.04	0.616
**Role functioning (RF2)**	Raw score<br>Final Score	1.41 +/- 0.67<br>86.17 +/- 22.34	1.37 +/- 0.61<br>87.59 +/- 20.40	0.664
**Emotional Functioning (EF)**	Raw score<br>Final Score	1.46 +/- 0.56<br>84.75 +/- 18.74	1.19 +/- 0.50<br>93.79 +/- 16.71	**<0.001**
**Cognitive Functioning (CF)**	Raw score<br>Final Score	1.24 +/- 0.58<br>91.84 +/- 19.31	1.19 +/- 0.52<br>93.62 +/- 17.22	0.359
**Social Functioning (SF)**	Raw score<br>Final Score	1.35 +/- 0.63<br>88.30 +/- 21.11	1.29 +/- 0.58<br>90.43 +/- 19.27	0.459
**SYMPTOM SCALES / ITEMS**				
**Fatigue (FA)**	Raw score<br>Final Score	1.50 +/- 0.67<br>16.55 +/- 22.21	1.35 +/- 0.49<br>11.82 +/- 16.45	0.110
**Nausea and Vomiting (NV)**	Raw score<br>Final Score	1.05 +/- 0.21<br>1.77 +/- 7.15	1.10 +/- 0.29<br>3.19 +/- 9.60	0.323
**Pain (PA)**	Raw score<br>Final Score	1.53 +/- 0.72<br>17.73 +/- 23.93	1.45 +/- 0.58<br>14.89 +/- 19.42	0.315
**Dyspnoea (DY)**	Raw score<br>Final Score	1.40 +/- 0.71<br>13.48 +/- 23.73	1.74 +/- 0.82<br>24.82 +/- 27.34	**0.017**
**Insomnia (SL)**	Raw score<br>Final Score	1.53 +/- 0.78<br>17.73 +/- 25.87	1.36 +/- 0.64<br>12.06 +/- 21.34	0.059
**Appetite loss (AP)**	Raw score<br>Final Score	1.21 +/- 0.62<br>7.09 +/- 20.78	1.21 +/- 0.51<br>7.09 +/- 16.93	1.000
**Constipation (CO)**	Raw score<br>Final Score	1.21 +/- 0.55<br>7.09 +/- 18.31	1.13 +/- 0.34<br>4.26 +/- 11.24	0.323
**Diarrhoea (DI)**	Raw score<br>Final Score	1.17 +/- 0.56<br>5.67 +/- 18.80	1.19 +/- 0.65<br>6.38 +/- 21.58	0.710
**Financial difficulties (FI)**	Raw score<br>Final Score	1.30 +/- 0.72<br>9.93 +/- 23.99	1.28 +/- 0.71<br>9.22 +/- 23.78	0.323
**QLQ – LC13**				
**SYMPTOM SCALES / ITEMS**				
**Dyspnoea (LCDY)**	Raw Score<br>Final Score	1.43 +/- 0.678<br>14.18 +/- 22.31	1.46 +/- 0.60<br>15.37 +/- 19.87	0.649
**Coughing (LCCO)**	Raw score<br>Final Score	1.66 +/- 0.64<br>21.99 +/- 21.17	1.57 +/- 0.62<br>19.15 +/- 20.55	0.351
**Haemoptysis (LCHA)**	Raw score<br>Final Score	1.04 +/- 0.29<br>1.42 +/- 9.72	1 +/- 0<br>0 +/- 0	0.323
**Sore mouth (LCSM)**	Raw score<br>Final Score	1.04 +/- 0.20<br>1.42 +/- 6.80	1.02 +/- 0.15<br>0.71 +/- 4.86	0.323
**Dysphagia (LCDS)**	Raw score<br>Final Score	1.17 +/- 0.52<br>5.67 +/- 17.47	1.17 +/- 0.48<br>5.67 +/- 16.03	1.000
**Peripheral neuropathy (LCPN)**	Raw score<br>Final Score	1.21 +/- 0.59<br>7.09 +/- 19.58	1.19 +/- 0.58<br>6.38 +/- 19.21	0.569
**Alopecia (LCHR)**	Raw score<br>Final Score	1.23 +/- 0.56<br>7.80 +/- 18.67	1.15 +/- 0.47<br>4.96 +/- 15.51	**0.044**
**Pain in chest (LCPC)**	Raw score<br>Final Score	1.21 +/- 0.46<br>7.09 +/- 15.44	1.15 +/- 0.36<br>4.96 +/- 12.0	0.371
**Pain in arm or shoulder (LCPA)**	Raw score<br>Final Score	1.30 +/- 0.59<br>9.93 +/- 19.55	1.30 +/- 0.66<br>9.93 +/- 21.89	1.000
**Pain in other parts (LCPO)**	Raw score<br>Final Score	1.51 +/- 0.86<br>17.02 +/- 28.55	1.47 +/- 0.78<br>15.60 +/- 25.87	0.323

Table S2: Full QOL Results (RATS vs VATS vs Thoracotomy)

Scale	Score	Thoracotomy	VATS	RATS	p-value
**QLQ-LC30**					
**GLOBAL HEALTH STATUS / QOL**					
**Global Health Status / QOL (QL2)**	Raw score<br>Final Score	5.00 +/- 1.58<br>66.67 +/- 26.35	4.97 +/- 1.43<br>66.24 +/- 23.76	5.43 +/- 1.06<br>73.76 +/- 17.63	0.176
**FUNCTIONAL SCALES**					
**Physical Functioning (PF2)**	Raw score<br>Final Score	1.78 +/- 0.75<br>73.94 +/- 25.11	1.72 +/- 0.68<br>75.95 +/- 22.63	1.42 +/- 0.51<br>86.10 +/- 16.90	**0.020**
**Role functioning (RF2)**	Raw score<br>Final Score	1.70 +/- 0.93<br>76.52 +/- 31.14	1.82 +/- 0.94<br>72.78 +/- 31.48	1.45 +/- 0.72<br>85.11 +/- 23.88	0.076
**Emotional Functioning (EF)**	Raw score<br>Final Score	1.61 +/- 0.68<br>79.76 +/- 22.60	1.66 +/- 0.80<br>77.99 +/- 26.56	1.22 +/- 0.51<br>92.79 +/- 17.02	**0.003**
**Cognitive Functioning (CF)**	Raw score<br>Final Score	1.45 +/- 0.52<br>84.92 +/- 17.40	1.58 +/- 0.65<br>80.77 +/- 21.67	1.22 +/- 0.52<br>92.55 +/- 17.31	**0.007**
**Social Functioning (SF)**	Raw score<br>Final Score	1.62 +/- 0.96<br>79.37 +/- 32.02	1.73 +/- 0.93<br>75.64 +/- 30.94	1.28 +/- 0.58<br>90.78 +/- 19.29	**0.015**
**SYMPTOM SCALES / ITEMS**					
**Fatigue (FA)**	Raw score<br>Final Score	2.28 +/- 0.78<br>42.68 +/- 25.97	2.02 +/- 0.90<br>34.04 +/- 30.00	1.46 +/- 0.57<br>15.37 +/- 19.04	**<0.001**
**Nausea and Vomiting (NV)**	Raw score<br>Final Score	1.20 +/- 0.37<br>6.82 +/- 12.24	1.20 +/- 0.42<br>6.75 +/- 14.00	1.13 +/- 0.32<br>4.26 +/- 10.69	0.540
**Pain (PA)**	Raw score<br>Final Score	1.64 +/- 0.54<br>21.21 +/- 17.95	1.72 +/- 0.87<br>23.84 +/- 28.95	1.49 +/- 0.59<br>16.31 +/- 19.81	0.264
**Dyspnoea (DY)**	Raw score<br>Final Score	2.27 +/- 0.98<br>42.42 +/- 32.82	2.10 +/- 0.90<br>36.71 +/- 30	1.77 +/- 0.81<br>25.53 +/- 27.11	**0.046**
**Insomnia (SL)**	Raw score<br>Final Score	1.82 +/- 1.10<br>27.27 +/- 36.57	1.75 +/- 0.99<br>24.89 +/- 33.11	1.45 +/- 0.77<br>14.89 +/- 25.83	0.164
**Appetite loss (AP)**	Raw score<br>Final Score	1.41 +/- 0.80<br>13.64 +/- 26.55	1.70 +/- 1.03<br>23.21 +/- 34.32	1.32 +/- 0.63<br>10.64 +/- 20.97	0.058
**Constipation (CO)**	Raw score<br>Final Score	1.50 +/- 0.86<br>16.67 +/- 28.64	1.49 +/- 0.78<br>16.46 +/- 26.08	1.21 +/- 0.51<br>7.09 +/- 16.93	0.088
**Diarrhoea (DI)**	Raw score<br>Final Score	1.38 +/- 0.80<br>12.70 +/- 26.82	1.18 +/- 0.48<br>5.98 +/- 15.89	1.23 +/- 0.70<br>7.80 +/- 23.27	0.403
**Financial difficulties (FI)**	Raw score<br>Final Score	1.24 +/- 0.70<br>7.94 +/- 23.34	1.19 +/- 0.54<br>6.49 +/- 17.97	1.26 +/- 0.71<br>8.51 +/- 23.54	0.863
**QLQ-LC13**					
**SYMPTOM SCALES / ITEMS**					
**Dyspnoea (LCDY)**	Raw score<br>Final Score	1.72 +/- 0.77<br>23.98 +/- 25.73	1.79 +/- 0.78<br>26.29 +/- 25.97	1.49 +/- 0.59<br>16.31 +/- 19.64	0.086
**Coughing (LCCO)**	Raw score<br>Final Score	2.10 +/- 0.83<br>36.51 +/- 27.70	1.85 +/- 0.82<br>28.44 +/- 27.23	1.6 +/- 0.65<br>19.86 +/- 21.6	**0.036**
**Haemoptysis (LCHA)**	Raw score<br>Final Score	1 +/- 0<br>0 +/- 0	1.03 +/- 0.23<br>0.89 +/- 7.70	1 +/- 0<br>0 +/- 0	0.639
**Sore mouth (LCSM)**	Raw score<br>Final Score	1.19 +/- 0.51<br>6.35 +/- 17.06	1.24 +/- 0.59<br>8.11 +/- 19.75	1.02 +/- 0.15<br>0.71 +/- 4.86	**0.047**
**Dysphagia (LCDS)**	Raw score<br>Final Score	1.01 +/- 0.30<br>3.17 +/- 10.03	1.19 +/- 0.48<br>6.22 +/- 16.16	1.19 +/- 0.50<br>6.38 +/- 16.5	0.699
**Peripheral neuropathy (LCPN)**	Raw score<br>Final Score	1.24 +/- 0.44<br>7.94 +/- 14.55	1.45 +/- 0.81<br>15.11 +/- 27.01	1.19 +/- 0.58<br>6.38 +/- 19.21	0.105
**Alopecia (LCHR)**	Raw score<br>Final Score	1.24 +/- 0.70<br>7.94 +/- 23.34	1.11 +/- 0.39<br>3.56 +/- 12.94	1.17 +/- 0.48<br>5.67 +/- 16.03	0.492
**Pain in chest (LCPC)**	Raw score<br>Final Score	1.52 +/- 0.51<br>17.46 +/- 17.06	1.29 +/- 0.63<br>9.78 +/- 21.06	1.19 +/- 0.40<br>6.38 +/- 13.26	0.073
**Pain in arm or shoulder (LCPA)**	Raw score<br>Final Score	1.52 +/- 0.75<br>17.46 +/- 24.99	1.47 +/- 0.86<br>15.77 +/- 28.79	1.4 +/- 0.80<br>13.48 +/- 26.61	0.837
**Pain in other parts (LCPO)**	Raw score<br>Final Score	1.75 +/- 1.02<br>25.00 +/- 33.98	1.93 +/- 1<br>31.02 +/- 33.25	1.62 +/- 0.95<br>20.57 +/- 31.51	0.233
